# Commitment of chondrogenic precursors of the avian scapula takes place after epithelial-mesenchymal transition of the dermomyotome

**DOI:** 10.1186/1471-213X-10-91

**Published:** 2010-08-31

**Authors:** Baigang Wang, Qin Pu, Raja De, Ketan Patel, Bodo Christ, Jörg Wilting, Ruijin Huang

**Affiliations:** 1 Department of Anatomy and Cell Biology, University of Goettingen, Kreuzbergring 36, 37075 Goettingen, Germany; 2 Institute of Anatomy and Cell Biology, University of Freiburg, Albertstrasse 17, 79104 Freiburg, Germany; 3 Institute of Anatomy, University of Bonn, Nussallee 10, 53115 Bonn, Germany; 4 School of Biological Sciences, University of Reading, Hopkins building, Whiteknights, Reading, RG6 6UB, Berkshire UK

## Abstract

**Background:**

Cells of the epithelially organised dermomyotome are traditionally believed to give rise to skeletal muscle and dermis. We have previously shown that the dermomyotome can undergo epithelial-mesenchymal transition (EMT) and give rise to chondrogenic cells, which go on to form the scapula blade in birds. At present we have little understanding regarding the issue of when the chondrogenic fate of dermomyotomal cells is determined. Using quail-chick grafting experiments, we investigated whether scapula precursor cells are committed to a chondrogenic fate while in an epithelial state or whether commitment is established after EMT.

**Results:**

We show that the hypaxial dermomyotome, which normally forms the scapula, does not generate cartilaginous tissue after it is grafted to the epaxial domain. In contrast engraftment of the epaxial dermomyotome to the hypaxial domain gives rise to scapula-like cartilage. However, the hypaxial sub-ectodermal mesenchyme (SEM), which originates from the hypaxial dermomyotome after EMT, generates cartilaginous elements in the epaxial domain, whereas in reciprocal grafting experiments, the epaxial SEM cannot form cartilage in the hypaxial domain.

**Conclusions:**

We suggest that the epithelial cells of the dermomyotome are not committed to the chondrogenic lineage. Commitment to this lineage occurs after it has undergone EMT to form the sub-ectodermal mesenchyme.

## Background

Epithelial and mesenchymal cells differ from each other in several aspects. Epithelial cells are connected to each other by cell surface contacts such as desmosomes and gap junctions, form dense cell layers resting on a basal lamina and exhibiting apical-basal polarity. In contrast, mesenchymal cells have no association with the basal lamina and usually do not possess elaborated adhesion complexes with neighbouring cells, which increase their migratory potential. Under certain physiological or pathological conditions epithelial cells lose their characteristics and undergo morphological changes to convert into mesenchymal cells, a biological process known as epithelial-mesenchymal transition (EMT) [1]. The process of EMT is reversible and the opposite mechanism converts mesenchymal cells into epithelia (mesenchymal-to-epithelial transition, MET).

EMT and MET play key roles not only during early embryonic development, for example during implantation of the embryo into the uterus, gastrulation and de-lamination of neural crest cells, but also during later developmental stages such as in somite development and organogenesis [2]. Somites are balls of epithelial cells, which arise from the paraxial mesoderm (mesenchyme) by means of MET. Later, the ventral half of each somite undergoes EMT to give rise to the sclerotome [3], while the dorsal half remains epithelial and forms the dermomyotome, source of the skeletal muscle, dermis [4], scapula blade in birds [5] and medial scapula border in mammals [6].

The avian dermomyotome in specific axial regions undergoes EMT to form sub-ectodermal mesenchyme which gives rise to scapula [7]. This process is controlled by signals from the ectoderm and the lateral plate mesoderm [7,8]. Furthermore only cells originating from the hypaxial but not the epaxial dermomyotome give rise to scapula [7]. It is still unknown when the fate of chondrogenic precursor cells is determined. To this purpose we performed quail-chick grafting experiments exchanging the epithelial or mesenchymal tissues between epaxial and hypaxial domains. Our results show that chondrogenic progenitor commitment takes place after the formation of sub-ectodermal mesenchyme.

## Methods

### Embryos

Fertilized White Leghorn chick eggs (*Gallus gallus*) and the Japanese quail (*Coturnix coturnix*) were purchased from a local breeder and were incubated at 37.8°C and 80% relative humidity. The embryos were staged according to Hamburger and Hamilton [9]. The quail embryos were staged according to Ainsworth et al (2010) [10].

### Semi-thin sections

Chick embryos at stages from HH-14 to HH-20 were fixed in 3.5% glutaraldehyde and 3.5% paraformaldehyde at 4°C overnight. After dehydration in an ethanol series (30%-100%), embryos were embedded in Epon at 60°C. 0.75 μm transverse semi-thin sections were made through the region of somite 20/21 and stained with methylene blue.

### Quail-chick grafting

The following grafting experiments from quail to chick embryos were performed at the level of somite 20/21:

(1) Transplantation of the epaxial dermomyotome with overlying ectoderm to hypaxial position at HH-16 (n = 24).

(2) Transplantation of the hypaxial dermomyotome with overlying ectoderm to epaxial position at HH-16 (n = 14).

(3) Transplantation of the epaxial SEM with overlying ectoderm to hypaxial position at HH- 20 (n = 24).

(4) Transplantation of the hypaxial SEM with overlying ectoderm to epaxial position at HH- 20 (n = 22).

All transplantations were performed with stage-matched quail and chick embryos. During transplantation, tissue of chick embryos was removed first, and then the desired quail tissue was isolated and transferred to the prepared position in the chick embryos. After a re-incubation periods of 5 hours to 6 days, the chimeras were harvested for further analysis using *in situ *hybridization, skeletal staining and immunohistochemistry. All embryos were younger than 10 days. Therefore, ethical approval of the experiments was not needed.

### Whole-mount *in situ *hybridization

Whole-mount *in situ *hybridization with *qPax1 *[11] was performed using a modified protocol of Nieto et al [12,13].

### Skeletal staining

Skeletal morphology was established by staining embryos for 24 hours with 0.015% Alcian blue in 80% ethanol and 20% acetic acid. The embryos were dehydrated in 100% ethanol for 24 h and then made transparent in 100% methylsalicylate [14].

### Immunohistochemistry

After skeletal staining, specimens were embedded in paraffin and transverse sections were taken through the operated region. Quail cells were detected with monoclonal QCPN-antibody (Developmental Studies Hybridoma Bank, Iowa City, IA, USA). A polyclonal anti-desmin-antibody (Sigma, Deisenhofen, Germany) was used to identify muscle cells. Secondary antibodies and colour reactions have previously been described [5]. On sections desmin appears in brown and the quail nuclei in blue. In some samples, only QCPN-antibody was used and stained with DAB-colour reaction. Using this protocol, quail nuclei appear brown. Finally, sections were counterstained with nuclear-fast-red (Sigma, Deisenhofen, Germany).

## Results

### Determining the time-window for EMT of scapula-forming dermomyotomes

**Figure 1:**
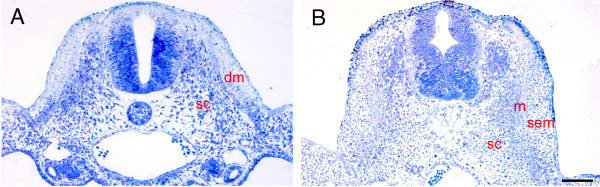
**Semi-thin section of somite 20/21**. A) The somite has formed the dorsally located epithelial dermomyotome (dm) and the ventrally located mesenchymal sclerotome (sc) at HH-16. B) At HH-20, the dermomyotome has undergone EMT to form myotome (m) and sub-ectodermal mesenchyme (sem) (Scale bar = 150 μm).

In this study we aimed to determine when scapular precursors in the dermomyotome are committed to a chondrogenic fate. We chose scapula-forming somite 20/21 for quail-chick grafting experiments. Firstly, it was necessary to study the morphology of scapula-forming somites to identify the stage when the scapular precursors in the dermomyotome form the sub-ectodermal mesenchyme (SEM). At HH-14, somite 20/21 is newly formed epithelial-walled spheroid (data not shown). At HH-16 it possesses two compartments, the dorsal dermomyotome, which has retained its epithelial structure (Fig. 1A), and the ventral sclerotome, a mesenchymal structure. Cells in dermomyotome exhibit typical epithelial characteristics, resting on a basal lamina and possessing apico-basal polarity. At HH-20 the central part of the dermomyotome has undergone EMT to form SEM, while the dorso-medial and ventro-lateral lips of dermomyotome remain epithelial (Fig. 1B). Based on these observations we performed heterotopic, interspecies transplantation of epithelial dermomyotome at HH-16, and of SEM at HH-20.

### Assessment of the experimental manipulations

**Figure 2:**
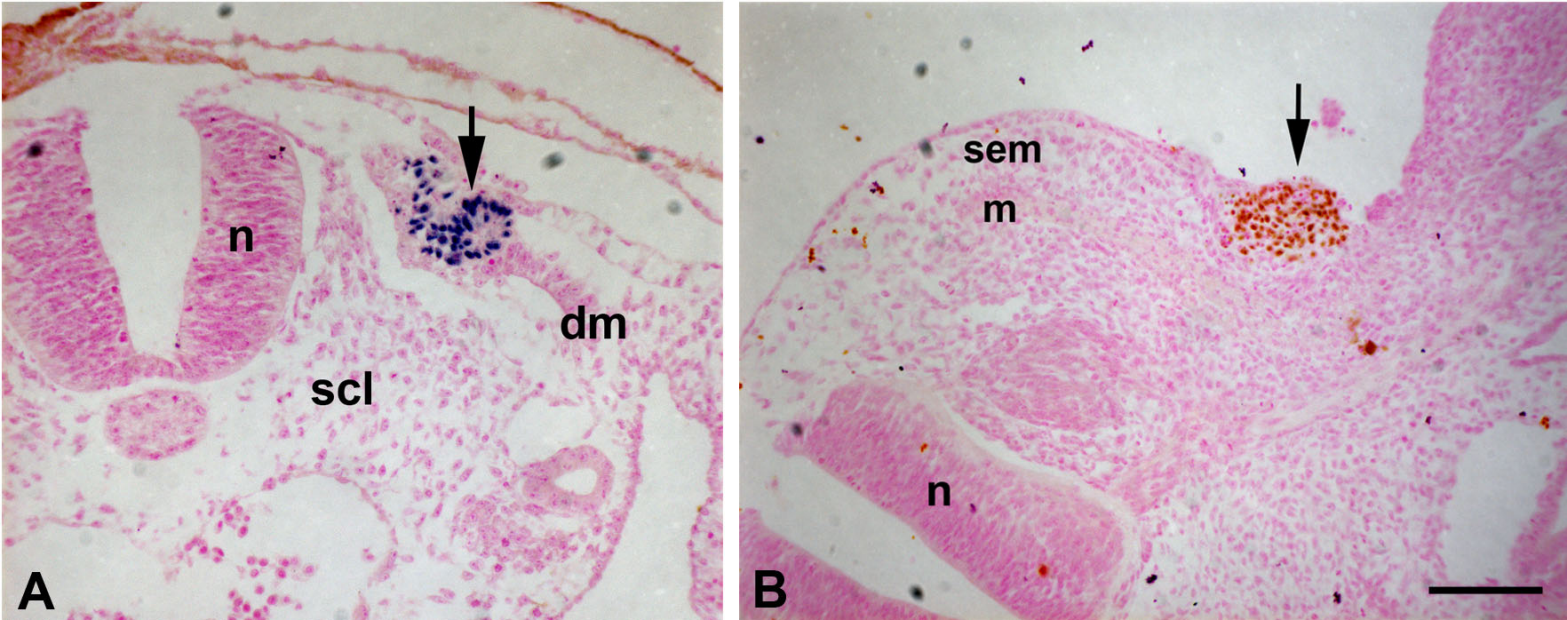
**Assessment of the grafting experiments**. After a re-incubation period of 5 hours the operated embryos were harvested, sectioned and stained with quail-specific QCPN antibody. A) Nuclear (blue dot) QCPN signal (black arrow) is confined to a small population of grafted dermomyotomal cells. B) Grafted SEM of quail origin (stained in brown) is located in the sub-ectodermal region (black arrow). dm, dermomyotome; m, myotome; n, neural tube; scl, sclerotome; sem, sub-ectodermal mesenchyme (Scale bar = 150 μm).

To evaluate whether the grafting experiments were performed cleanly, we harvested some operated embryos five hours after the transplantation of the epithelial dermomyotome and the SEM. Transverse sections were processed for immunohistochemistry with quail-specific QCPN-antibody. Quail hypaxial dermomyotome without any sclerotome cells survived and developed after being transplanted into the epaxial region in chick host (Fig. 2A). Epaxial SEM of quail origin was situated in the hypaxial region in chick after a re-incubation period of five hours (Fig. 2B). It is noteworthy that after 5 hours of re-incubation the quail ectoderm had almost disappeared and was replaced by chick ectoderm (Fig. 2A, B) and after longer re-incubation periods, it was difficult to identify altogether. Since the ectoderm consists of only a single cell layer it is easily damaged during the operation, and may degenerate.

### Epaxial epithelial dermomyotome grafted to the hypaxial position gives rise to chondrocytes

**Figure 3:**
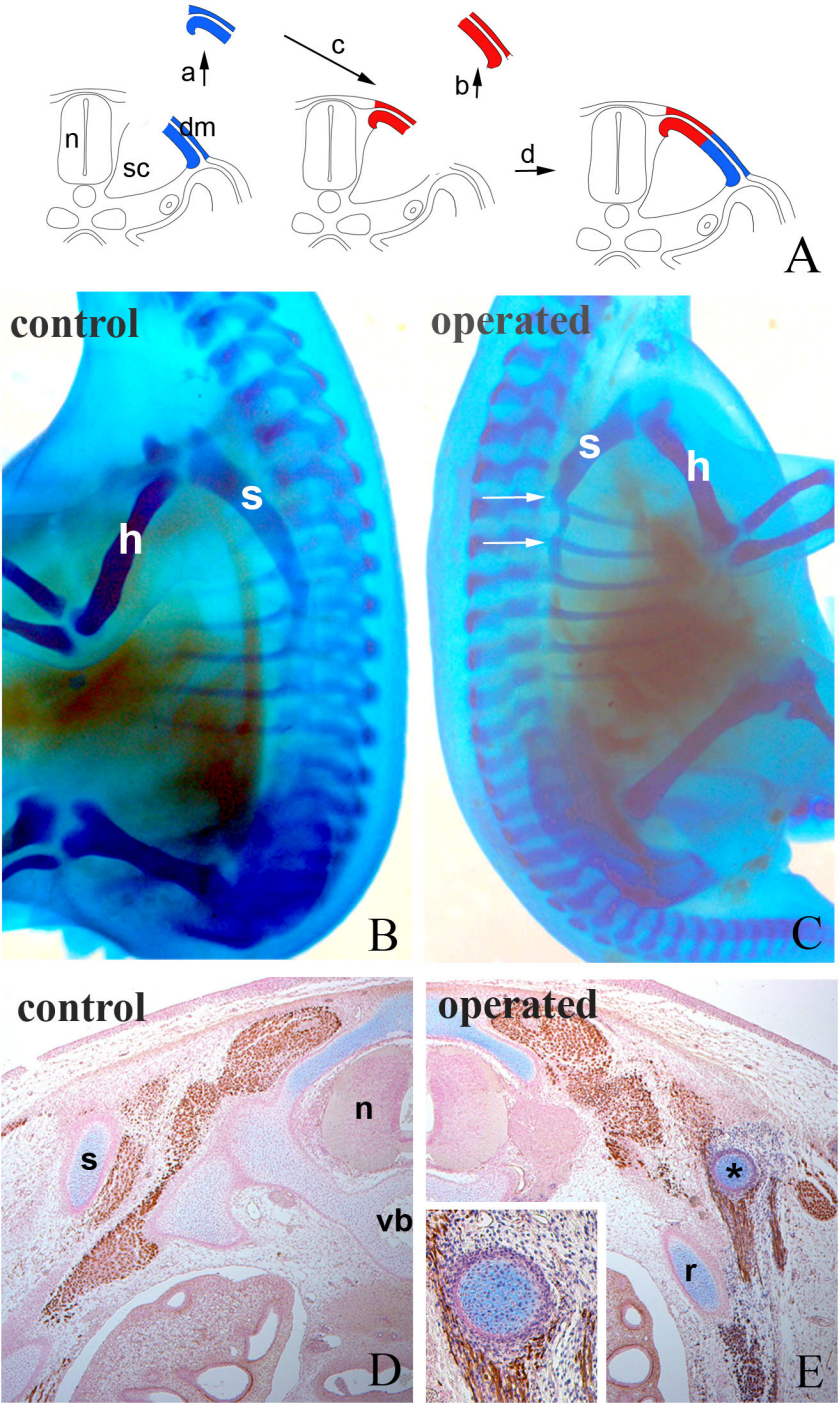
**Epaxial epithelial dermomyotome grafted to the hypaxial domain gives rise to chondrocytes**. A) Schematic illustration of the grafting procedure. Quail epaxial dermomyotome (blue) of somite 20/21 was transplanted to the hypaxial domain of chick embryo (red). dm, dermomyotome; n, neural tube; sc, sclerotome. B and C) Skeletal staining after six days of re-incubation. B) Control side shows normal scapula (s). C) Operated side shows a slim skeletal element at the level of the second thoracic segment (between arrows) connected to the remaining chick scapula (s). Vertebral column, rib and humerus (h) are normal. D and E) Immunohistochemistry on transverse sections through the operated region. D) Control side shows normal scapula (s), vertebral body (vb) and neural tube (n). E) Operated side exhibits a scapula of quail origin (black asterisk) and a normal rib (r) of chick origin. Insert in E) shows higher magnification of scapula with QCPN-positive cells.

To assess whether cells are committed to the chondrogenic lineage while in the epithelial dermomyotome, transplantations between scapula-forming and non-scapula-forming somite compartments were performed. We have shown by homotopic transplantation of epaxial and hypaxial dermomyotome compartments that the avian scapular blade derives from the hypaxial dermomyotome of somites 17-24, whereas the epaxial dermomyotome never contributes to the scapula [5,7]. Therefore we firstly transplanted quail epaxial dermomyotome at HH-16 to chick hypaxial domain of somite 20/21 (Fig. 3A). A total of 24 transplantations were carried out. After six days of re-incubation 22 host embryos were harvested. After Alcian blue staining, 19 embryos showed a slim cartilaginous element which was connected to the chick scapula (Fig. 3B, C). Only three embryos failed to show any cartilaginous anlagen of the scapula. To clarify whether this skeletal element was of quail origin, 10 specimens were transversely sectioned through the operated area and stained with quail-specific QCPN-antibody. In 6 samples, quail cells were found after the immunohistochemistry on paraffin sections. Quail cells were identified in the scapula-like cartilage, perichondrium, scapula-attaching muscles and intercostal muscles, all of which are derivatives of the hypaxial dermomyotome (Fig. 3D, E). Quail cells were not found in ribs, showing that our transplant did not contain any sclerotomal cells [15].

In our previous study [7], the hypaxial dermomyotomal epithelium was homotopically grafted into the hypaxial domain. This experiment can serve as a control for this study. In most cases, the host embryo displayed a thin scapula segment in the operated region. In this scapula, quail cartilage tissue was found. This indicated that the manipulation can influence the scapula morphogenesis. However, the differentiation programme of the grafted dermomyotomal cells was not altered.

We conclude that in the new environment, the epaxial dermomyotome acquires the potential to form hypaxial derivatives, such as scapula.

### Hypaxial epithelial dermomyotome grafted to the epaxial position does not form chondrocytes

**Figure 4:**
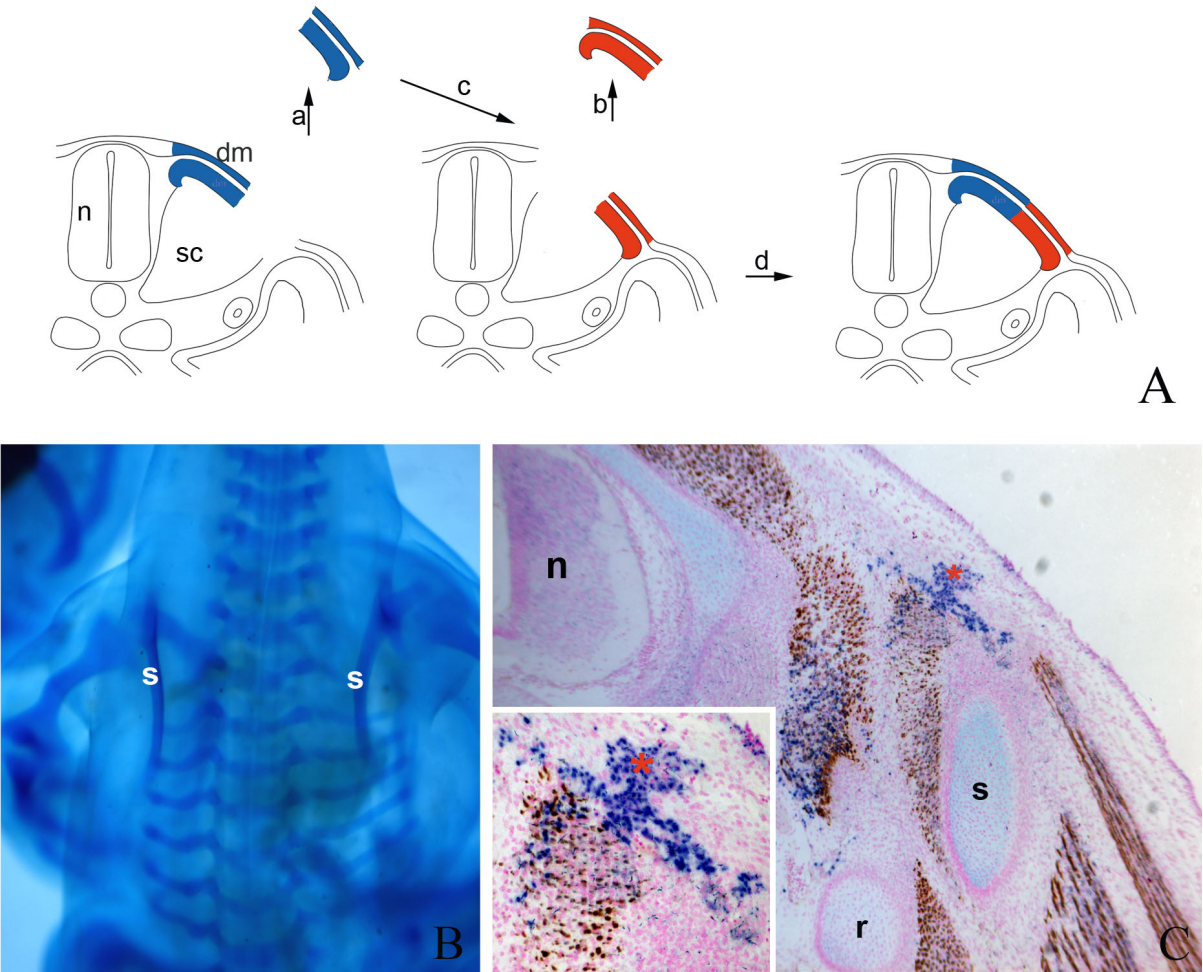
**Hypaxial epithelial dermomyotome grafted to the epaxial domain does not form chondrocytes**. A) Schematic illustration of the grafting procedure. Quail hypaxial dermomyotome (blue) of somite 20/21 was transplanted to the epaxial domain of chick embryo (red). dm, dermomyotome; n, neural tube; sc, sclerotome. B) Whole-mount skeletal staining showed a normal chick scapula (s) in the operated region (right). C) Immunohistochemistry on transverse sections through the operated region. Operated side exhibits a cluster of quail cells (red asterisk) giving rise to muscles and connective tissue. n, neural tube; r, rib; s, scapula. Insert in C) shows higher magnification of the grafted QCPN-positive quail cells (red asterisk).

Since the epaxial dermomyotomal epithelium possesses developmental plasticity, we further addressed the question whether the hypaxial compartment can generate cartilage in an epaxial domain. To answer this question, we transplanted quail hypaxial dermomyotomal epithelium at HH-16 into chick epaxial domain at the level of somite 20/21 (Fig. 4A). A total of 14 transplantations were performed and 13 embryos survived until the required stage of development. After six days of re-incubation the embryos were harvested and subjected to skeletal staining. None exhibited any ectopic cartilage in the epaxial domain, and only the normal chick scapula was visible (Fig. 4B). Immunohistochemical staining of transverse sections through the operated region showed quail cells in the epaxial domain. These cells contributed to autochthonal muscles and dermis, but not to cartilage (Fig. 4C). These results are in line with those after the homotopic transplantation of the epaxial dermomyotome (as a control) in our previous work [7].

This observation confirms that scapular precursors in the dermomyotome are not committed to the chondrogenic lineage. They do not form cartilage but develop according to local cues when placed in the epaxial domain.

### Epaxial SEM grafted to the hypaxial domain cannot generate chondrocytes

**Figure 5:**
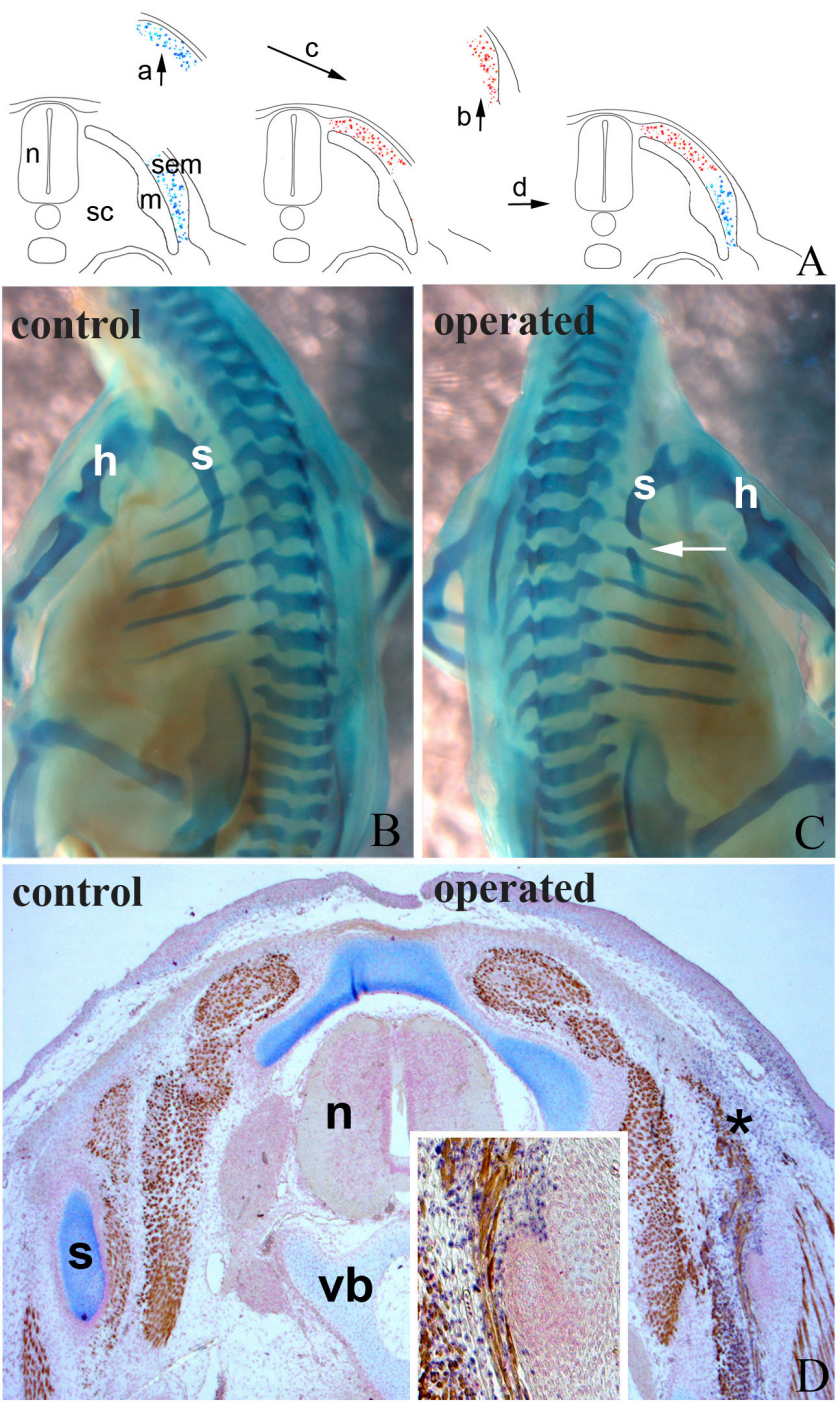
**Epaxial SEM grafted to the hypaxial domain cannot generate chondrocytes**. A) Schematic illustration of the grafting procedure. Quail epaxial SEM (blue) at the level of somite 20/21 was transplanted to the hypaxial domain of chick embryo (red). m, myotome; n, neural tube; sc, sclerotome; sem, sub-ectodermal mesenchyme. B and C) Skeletal staining after five days of re-incubation. B) Control side shows normal scapula (s) and humerus (h). C) Operated side reveals a missing segment of the scapula (s) as indicated by the white arrow. D) Immunohistochemistry on transverse sections through the operated region. Quail cells (black asterisk) are found in dermis and inter-muscular connective tissue. n, neural tube; s, scapula; vb, vertebral body.

Since chondrogenic scapular precursors are not yet committed in the epithelial state, we determined when this choice is made. A subpopulation of dermomyotome cells become mesenchymal and migrate towards the ectoderm to form SEM. We divided this mesenchyme into epaxial and hypaxial compartments and, using HH- 20 embryos, transplanted quail epaxial SEM to the hypaxial position of chick embryos at the level of somite 20/21 (Fig. 5A). A total of 24 transplantations were performed and 20 chimeras survived until day 9/10 and were harvested for skeletal staining followed by immunohistochemistry. In most specimens a segment of the scapula was missing. Ribs and vertebral column were normal in all cases (Fig. 5B, C). Grafted quail cells were found in 6 host embryos. In the operated region, scattered quail cells were found in dermis and intermuscular connective tissue, but none in the cartilage (Fig. 5D). A few quail cells were present in muscles, probably because the transplants contained myotome cells.

To test to the robustness of our transplantation process, quail SEM was grafted from the hypaxial domain of a quail donor into the same domain of a chick host (n = 6). In 4 out of 6 chimeras, quail cells were found in the scapula cartilage (Additional file 1). In 2 out of 6 samples, a segment of the scapula was missing and few quail cells could be seen. These results indicate that the manipulation may result in malformation in case of failure of engraftment. However, the homotopically grafted quail cells do give rise to cartilage tissue.

Our experiments demonstrate that normal scapula development is disturbed after replacement of hypaxial SEM with epaxial SEM.

### Hypaxial SEM grafted to the epaxial domain generates chondrocytes

**Figure 6:**
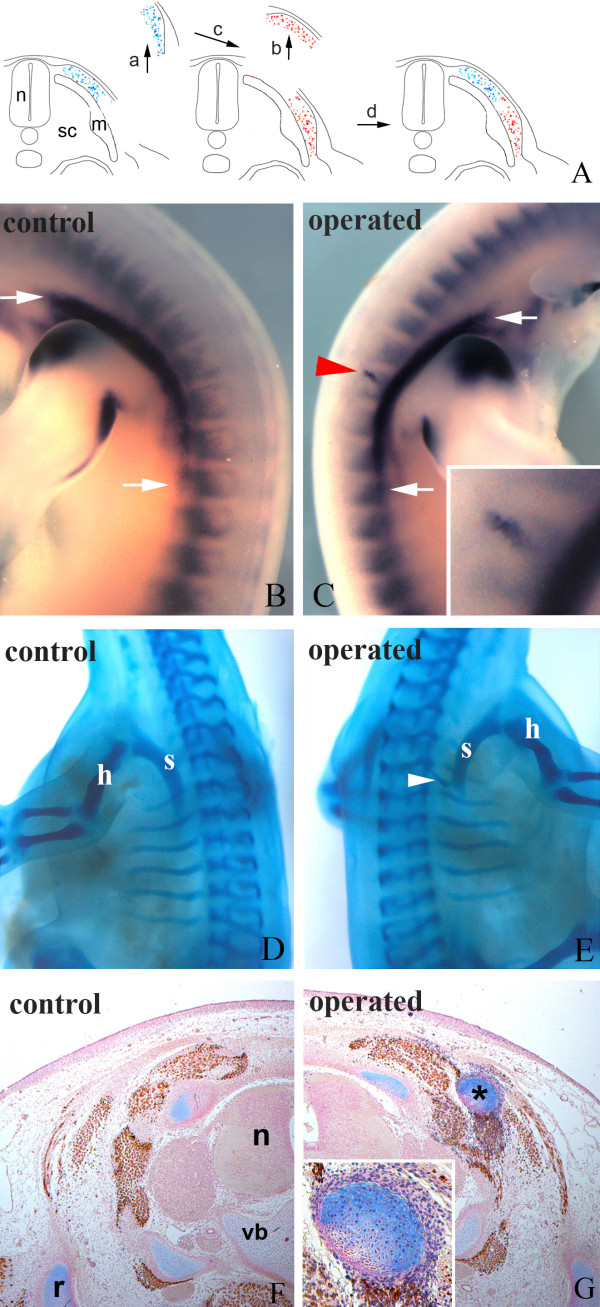
**Hypaxial SEM grafted to the epaxial domain generates chondrocytes**. A) Schematic illustration of the grafting procedure. Quail hypaxial SEM (blue) at the level of somite 20/21 was transplanted to the epaxial domain of chick embryo (red). m, myotome; n, neural tube; sc, sclerotome; sem, sub-ectodermal mesenchyme. B and C) *In situ *hybridization with *qPax1 *after two days of re-incubation. B) Control side shows normal *Pax1 *expression pattern in scapula anlagen (region between white arrows). C) Abnormal expression of *Pax1 *in epaxial domain (red arrowhead) in a central, operated region of the chick *Pax1*-positive scapula anlagen (between white arrows). D and E) Skeletal staining after five days of re-incubation. D) Control side shows normal scapula (s) and humerus (h). E) Operated side shows an ectopic cartilaginous element besides the normal scapula (white arrowhead). F and G) Immunohistochemistry on transverse sections through the operated region. F) Control side shows normal skeletal elements. n, neural tube; r, rib; vb, vertebral body. G) Operated side shows an ectopic cartilaginous element (black asterisk) of quail origin in epaxial position. Insert in G) shows higher magnification of QCPN-positive cells in the ectopic cartilage.

Since the epaxial SEM has lost its ability to form hypaxial derivatives, we then investigated whether hypaxial SEM generates ectopic cartilage in an epaxial position. To this end, we transplanted quail hypaxial SEM to the epaxial domain in chick embryos at level of somite 20/21 (Fig. 6A). A total of 22 transplantations were carried out. After two days of re-incubation, eight specimens were harvested and prepared for *qPax1 in situ *hybridization. After five days of re-incubation, the remaining embryos were prepared for skeletal staining and immunohistochemistry. After *in situ *hybridization, all of the eight embryos showed ectopic expression of the scapular marker *Pax1 *in epaxial position (Fig. 6B, C) indicating that grafted quail cells had initiated chondrogenesis. The skeleton of the other 14 operated embryos exhibited ectopic cartilage aside the normal scapula (Fig. 6D, E). Immunohistochemistry confirmed that the chondrocytes and the perichondrium were of quail origin (Fig. 6F, G). In addition, quail cells were located in dermis and intermuscular connective tissue. Some autochthonal muscles were of quail origin, most likely because of the presence of a few myotomal cells in the transplanted tissue. In summary, our results show that chondrogenic scapular precursor cells are committed after they have undergone epithelial-mesenchymal transition.

## Discussion

In the present study we aimed to identify when dermomyotomal cells are committed to the formation of cartilage. Our results, based on a series of quail-chick transplantation experiments, show that these precursors are not committed as long as they are situated in the dermomyotome, but are committed to form cartilage after going through EMT to generate the sub-ectodermal mesenchyme (SEM).

Developmental plasticity of newly formed epithelial somites has been demonstrated in previous studies. Experiments involving the dorsal-ventral rotation of epithelial somites have shown that cells develop according to their new position [16,17]. These results demonstrate that cells of the epithelial somites lack intrinsic patterning information. Instead, their commitment relies on information imparted by local signalling centres. The results presented here provide another example for the plasticity of the epithelial somitic compartment, the dermomyotome. The fact that hypaxial dermomyotome grafted into epaxial position forms muscles instead of scapular cartilage strongly suggests that hypaxial dermomyotome is not committed at this stage, and is competent to generate epaxial derivatives in the new environment. This led us to the hypothesis that epaxial dermomyotome will differentiate into scapula when grafted to a hypaxial position. Generation of cartilaginous elements made up of transplanted quail donor cells proved this hypothesis to be correct. We further investigated whether scapula precursors are committed to their chondrogenic fate once they form the SEM. Hypaxial SEM grafted into an epaxial position generated cartilage, whereas the reciprocal transplantation of epaxial SEM into the hypaxial domain did not produce chondrocytes. This demonstrates that chondrogenic scapular precursor cells are committed once SEM has formed as a consequence of EMT of the epithelial dermomyotome.

Our results showing the demise of the donor ectoderm and its replacement by host ectoderm, followed by normal dermomyotome development, suggest that regenerated ectoderm develops characteristics associated to its normal position along the medio-lateral axis. In a previous study, we showed that signals from the overlying ectoderm are essential for scapula development [8]. The overlying ectoderm probably functions in two phases. In the early stages, it is responsible for maintaining the epithelial structure of the dermomyotome by secreting Wnt6. Wnt6 signal from the ectoderm, which may act in the chick via canonical and non-canonical pathways during neural crest development [18], maintains the epithelial structure of the dermomyotome. Thereby it regulates the expression of the epithelial marker *paraxis *[19]. In the later stages, decrease of Wnt6 in ectoderm [20] facilitates EMT of the dermomyotome, as a consequence of which the scapular precursors migrate to the sub-ectodermal space to get committed to the chondrogenic lineage by local cues. Disturbed EMT in dermomyotome caused by a prolongated epithelial status may result in a defect scapula. This has been suggested by the observation that overexpression of *carboxypeptidase Z *(CPZ), a secreted enzyme which promotes Wnt signalling, results in up-regulation of the Wnt-induced epithelial marker *Pax3 *in the hypaxial dermomyotome, down-regulation of *Pax1 *in the scapular anlage and loss of scapula [21]. The EMT of the scapular precursor cells in dermomyotome and their commitment in the sub-ectodermal space might be two independent processes. We suggest that cells undergoing EMT are able to respond to pro-chondrogenic signalling which they were unable to do in the epithelial state. It is possible that alterations of the cell membrane, especially modifications of receptors and cell adhesion molecules change not only epithelial cell characteristics but also initiate gene transcription, which may permit cells to respond to local cues. This can be exemplified by the dual role of β-Catenin during EMT. β-Catenin in cell membranes is associated with cell-cell contact in both normal epithelium and non-invasive tumours. On the other hand, in cells undergoing EMT, β-Catenin is translocated from the cell membrane to the nucleus and functions together with the DNA-binding factor TCF as a transcriptional activator [22,23]. The somitic scapular precursor cells express *Pax1*, which has first been identified as a sclerotomal marker gene [24-26]. In response to notochord-derived sonic hedgehog, *Pax1 *is expressed in the ventral half of the somites shortly before their de-epithelialization [27], thereby regulating development of the vertebral column [28]. In contrast, in the scapular precursor cells *Pax1 *is activated a considerable time after initiation of EMT of the dermomyotome. *Pax1 *expression can be seen in the scapular anlagen only from HH-26 onward. Our transplantations of SEM were carried out at HH-20 and generated Pax1-positive cells when hypaxial SEM was grafted into an epaxial position. Our results show that commitment of scapular precursor cells is controlled by genes other than *Pax1*.

Local cues for commitment of the scapular precursor cells may come from the neighbouring structures like myotome and somatopleure. FGF signaling from myotome increases the proliferation of chondrogenic precursor cells in sclerotome, but has no dramatic effect on dermomyotome [29]. Furthermore, FGF signalling probably is not involved in commitment of the scapula precursor cells since inhibition of FGF receptor-1 does not affect scapula blade development [30]. Since scapula precursor cells in SEM lie adjacent to the lateral somatopleure and somatopleure-derived BMP signalling is required for scapula development [7], we propose that scapular precursor cells get committed in the sub-ectodermal space by BMP signalling from the lateral somatopleure.

## Conclusions

We show that in the epithelial dermomyotome cells are not committed to the chondrogenic lineage. Commitment to chondrogenic cell fate occurs after the formation of sub-ectodermal mesenchyme.

## Authors' contributions

BW, QP and RH carried out the quail-chick grafting experiments. BW drafted the manuscript. KP and JW were engaged in discussion of the results and drafting of the manuscript. BW, RD and QP performed whole-mount *in situ *hybridization, skeletal staining and immunohistochemistry. RH and BC designed the study. All authors read and approved the final manuscript.

## Supplementary Material

Additional file 1 **Control experiment**. As control experiment the quail hypaxial SEM at the level of somite 20/21 was transplanted into the same region in chick. After five days of re-incubation, operated embryos were prepared for skeletal staining and immunohistochemistry with quail specific QCPN-antibody. A) Control side shows a normal scapula (s), vertebral column and ribs. B) Operated side shows a deformed but differentiated cartilaginous element in the scapular (s) anlagen (white arrow). C) Immunohistochemistry shows that the cartilaginous element is of quail origin (quail nuclei: brown). Insert in C) shows higher magnification of QCPN-positive cells in the scapular anlagen. Click here for file
