# Supplementary figures and images for: Commitment of chondrogenic precursors of the avian scapula takes place after epithelial-mesenchymal transition of the dermomyotome
Source: BMC Dev Biol. 2010 Aug 31;10:91. doi: 10.1186/1471-213X-10-91 (PMC2936895; doi:10.1186/1471-213X-10-91)

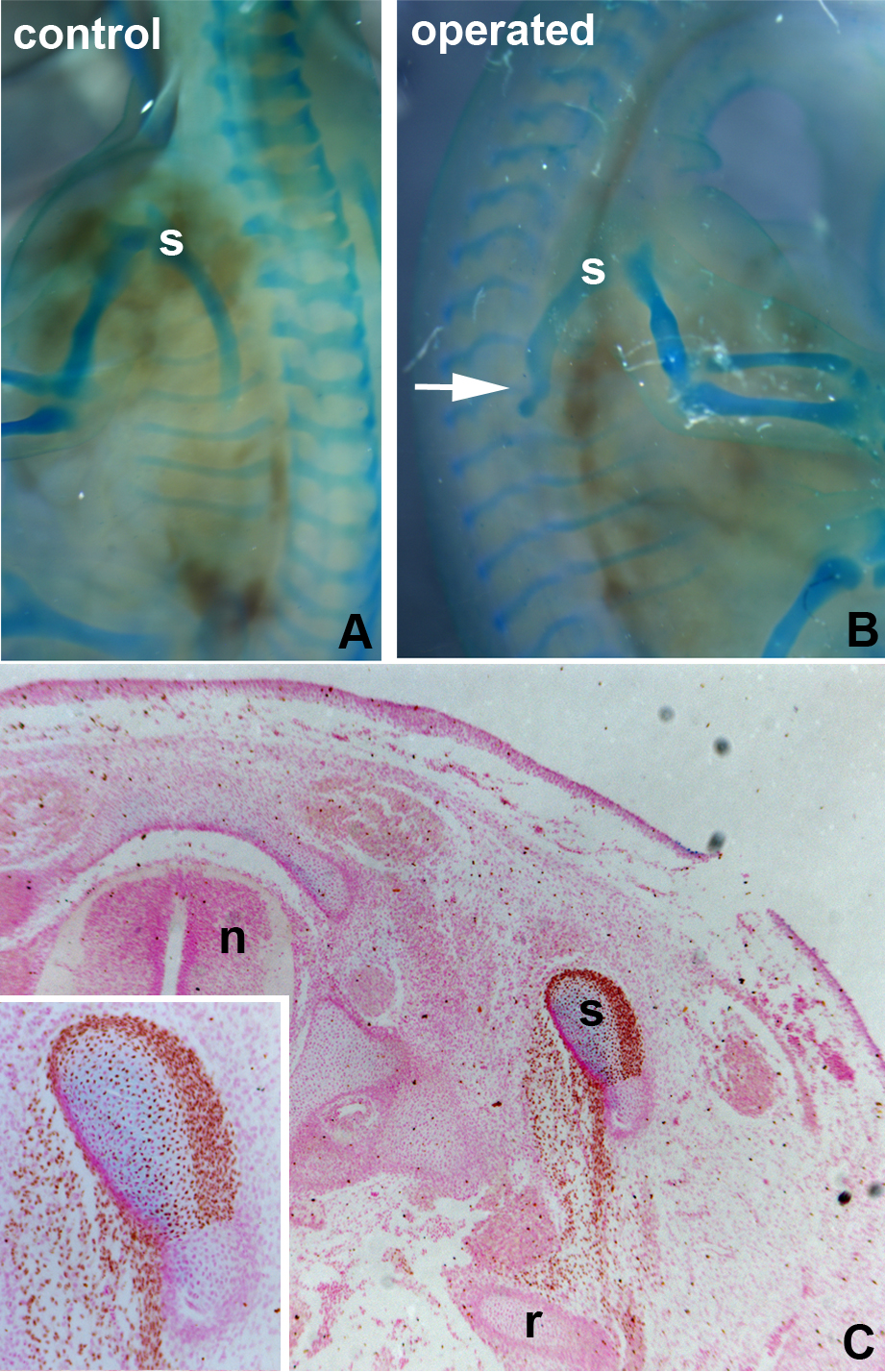

Supplement: Additional file 1 — Control experiment. As control experiment the quail hypaxial SEM at the level of somite 20/21 was transplanted into the same region in chick. After five days of re-incubation, operated embryos were prepared for skeletal staining and immunohistochemistry with quail specific QCPN-antibody. A) Control side shows a normal scapula (s), vertebral column and ribs. B) Operated side shows a deformed but differentiated cartilaginous element in the scapular (s) anlagen (white arrow). C) Immunohistochemistry shows that the cartilaginous element is of quail origin (quail nuclei: brown). Insert in C) shows higher magnification of QCPN-positive cells in the scapular anlagen. [file 1471-213X-10-91-S1.JPEG]
